# A two-domain regulatory literacy instrument among Saudi pharmacy interns: a single-center pilot assessment under vision 2030

**DOI:** 10.3389/fmed.2026.1926745

**Published:** 2026-09-23

**Authors:** Mohammed Ghazi M. Almutairi, Sadeem Almohaimeed, Aishah Alazzaz, Nadia Alharbi, Aryam Almutairi, Ghadi Alrasheedi

**Affiliations:** 1 Department of Pharmacy Practice, College of Pharmacy, Qassim University, Buraidah, Saudi Arabia; 2 Pharmacy Practice Department, College of Pharmacy, Qassim University, Saudi Arabia, Buraydah, Saudi Arabia

**Keywords:** pharmacy education, pharmacy interns, pharmacy law, procedural competency, declarative competency, regulatory literacy, Saudi Arabia, SFDA

## Abstract

**Background:**

Saudi Arabia accelerates the nationalization of the pharmacy workforce under Vision 2030, making regulatory proficiency paramount for ensuring patient safety and institutional compliance. While foundational jurisprudence is integrated into Doctor of Pharmacy (PharmD) curricula, a significant knowledge-to-action gap often persists regarding the procedural execution of statutory mandates.

**Objectives:**

This pilot study aimed to develop a bilingual, two-domain regulatory literacy instrument and to compare declarative (basic) vs. procedural (operational) regulatory literacy of final-year pharmacy interns.

**Methods:**

A cross-sectional pilot assessment was conducted among final-year PharmD interns (*N* = 40). An 11-item two-domain instrument assessed regulatory competencies across declarative (6 items) and procedural (5 items) domains. Statistical dependence was controlled by utilizing McNamar's test to evaluate performance variations across domains within the same cohort. The predictive validity of self-perceived literacy was analyzed using Fisher's exact test.

**Results:**

The cohort demonstrated high proficiency within the declarative sub-domain (80.0% overall success rate), with 95% correctly identifying pharmacy manager nationality mandates. Conversely, a critical deficiency emerged within the procedural sub-domain (23.5% overall success rate). Alarmingly, 50% (*n* = 20) misidentified the United States Food and Drug Administration as the local market authorization body, and (0%, *n* = 0) were aware of commercial non-promotional value caps. At the participant level, 80.0% passed the declarative sub-domain (95% CI: 64.4%–90.9%), whereas only 22.5% passed the procedural sub-domain, confirming a highly significant operational performance gap (95% CI: 10.8%–38.5%; *χ*^2^ = 21.04, df = 1, *p* < 0.0001). Self-perceived literacy strongly predicted objective outcomes (Fisher's exact test, *p* < 0.0001). However, this calibration exposed a profound systemic under preparedness.

**Conclusion:**

These exploratory findings suggest a notable vulnerability within the evaluated cohort, where strong declarative knowledge masks deficits in procedural execution. To support practice-ready graduates and mitigate compliance risks under Saudi Vision 2030, a transition from theoretical jurisprudence to simulated operational training frameworks is recommended within undergraduate pharmacy curricula.

## 1 Introduction

The legislative framework governing pharmaceutical establishments in the Kingdom of Saudi Arabia serves as a critical pillar for public health, institutional accountability, and patient safety (1, 2). Primary regulatory oversight is shared by the Saudi Food and Drug Authority (SFDA) and the Ministry of Health (MOH) (1, 3). It is critical to establish that while the MOH retains health facility licensure and high-level operational oversight, the SFDA strictly governs product registration, pharmacovigilance, and commercial promotional conduct. Foundational regulatory parameters are rooted in the Law of Practicing Healthcare Professions (Royal Decree No. M/59), which defines the ethical and professional boundaries for practitioners (1). Modern administrative governance is provided by the Implementing Regulations of the Law of Pharmaceutical and Herbal Establishments and Products (Royal Decree No. M/108) and the Saudi Code of Conduct for Promotional Practices of Pharmaceutical and Herbal Products (4, 5). The educational significance of regulatory literacy is highlighted by the Saudi Commission for Health Specialties, which allocates 20% of the Saudi Pharmacy Licensing Examination blueprint to social, administrative, and legal sciences (3).

Despite the clear professional importance of these regulations, emerging national literature suggests a critical knowledge-to-action (KTA) gap in regulatory literacy among graduating pharmacy students (6, 7). To analyze this deficit, regulatory literacy must be divided into two distinct domains: declarative (basic) knowledge, which encompasses the conceptual understanding of statutory prohibitions and ethical boundaries, and procedural (operational) knowledge, which dictates the administrative execution of these frameworks within daily management settings (6). National mapping of Saudi undergraduate PharmD programs reveals that regulatory concepts are often relegated to a single, 2-credit-hour theoretical course near the final year of study (7). Consequently, pharmacy students frequently transition to experiential clerkships and subsequent professional practices without acquiring a sufficient operational understanding of the regulatory workflows mandated by sovereign authorities (8, 9). Historically, a national cross-sectional evaluation by Alkayyal, Cheema, and Hadi established that more than 64% of pharmacy students acknowledged not knowing how to report adverse drug reactions (8). This structural gap represents an urgent challenge as the Saudi pharmacy workforce undergoes rapid nationalization (Saudization) aligned with Saudi Vision 2030. Emerging pharmacists are increasingly expected to assume immediate, unsupervised legal liabilities as pharmacy managers and institutional compliance officers. Thus, a robust, action-oriented command of national regulations is vital to avoid severe structural non-compliance, financial liabilities, and compromises to patient safety.

On a global scale, the disconnect between theoretical legal awareness and operational execution mirrors challenges documented within international health professions education. Jurisprudence in North American and European cohort studies indicate that while pharmacy interns routinely demonstrate high proficiency in traditional classroom-based regulatory evaluations, their performance drops when navigating real-world, non-scripted administrative compliance parameters during experiential clinical rotations (10, 11).

This study addresses this empirical gap by conducting a single-center pilot assessment of pharmacy regulatory literacy using a two-domain instrument designed to differentiate between declarative and procedural compliance capacities. By evaluating an emerging cohort of final-year pharmacy interns, this research quantifies the operational gap in pharmaceutical law, highlights critical curricular vulnerabilities, and provides an empirical baseline for national educational transformation.

## 2 Methods

### 2.1 Study design and participant selection

This cross-sectional study was conducted during the 2025–2026 internship cycle. Inclusion criteria were strictly limited to final-year Doctor of Pharmacy (PharmD) students currently enrolled in the institutional internship program at Qassim University, representing the emerging regional workforce. Recruitment was facilitated via an official institutional invitation broadcast through secure university portals and student email directories by an authorized representative. A convenience sampling strategy was utilized, capturing a final sample of 40 participants out of the eligible pool of 100 interns, yielding a 40% response rate (Figure 1).

**Figure 1:**
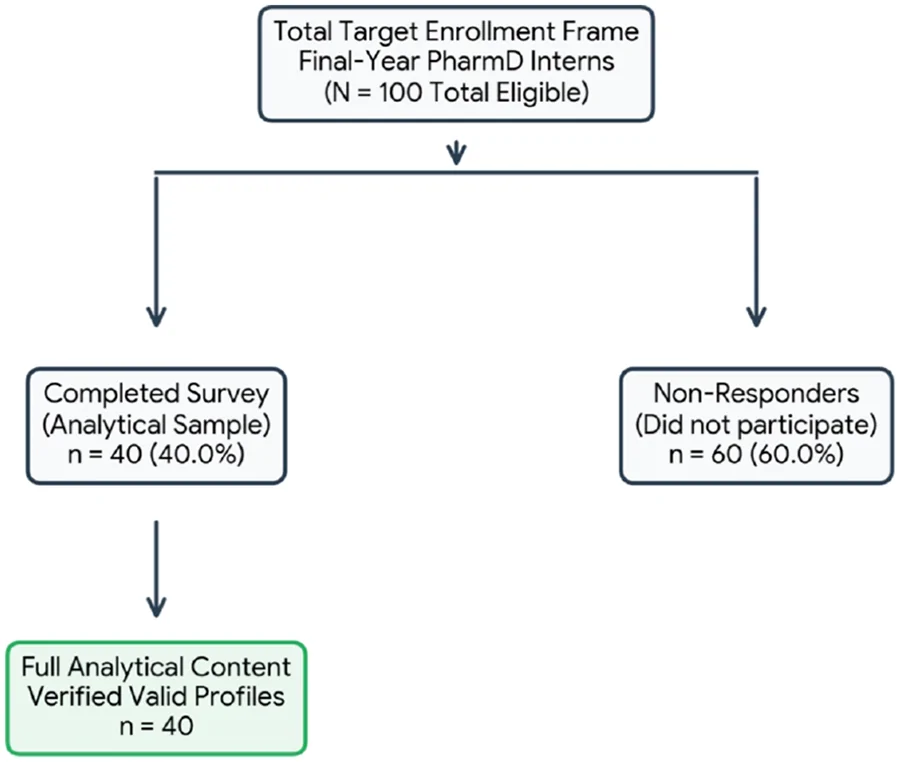
Strengthening the reporting of observational studies in epidemiology participant flow diagram.

### 2.2 Primary objective

To quantify and compare the levels of basic declarative versus operational procedural regulatory literacy among a cohort of final-year PharmD interns, explicitly mapping the knowledge-to-action (KTA) gap in pharmaceutical law.

### 2.3 Secondary objective

To evaluate the predictive validity of interns' self-perceived regulatory literacy against their objective assessment performance to determine the reliability of self-assessment frameworks in identifying systemic compliance risks.

### 2.4 Instrument development and content verification

A structural, bilingual (English/Arabic), web-administered survey instrument was custom-developed to measure discrete regulatory elements (see Supplementary file 1). To establish objective content validity, a multi-disciplinary expert panel comprising three experts (including two senior academic pharmacy faculty members and one specialized regulatory compliance officer) evaluated an initial pool of 12 preliminary items for clarity, contextual relevance, and appropriate domain assignment to ensuring exact alignment with Royal Decree No. M/108 and the Saudi Code of Conduct for Promotional Practices (4, 5). During this panel evaluation phase, one item within the operational domain was deleted based on the recommendation of an experienced pharmacy faculty member serving as a regulatory compliance reviewer, who noted that the specific question topic fell outside the expected familiarity threshold for final-year interns. This qualitative pruning ensured the instrument remained strictly representative of baseline graduate expectations, yielding the finalized 11-item instrument (6 declarative items, 5 procedural items) utilized for active data collection. The resulting Item-Content Validity Index (I-CVI) for the final items ranged from 0.67 to 1.00, yielding an overall Scale-Content Validity Index (S-CVI/Ave) of 0.91, which significantly exceeds the established psychometric threshold of 0.80 for robust content validity mapping. Face validity and linguistic clarity were piloted within the regional cohort, with 100% of respondents confirming clear instructional parameters. The instrument was partitioned into two primary functional components. The first section gathered socio-demographic indicators, legal source utilization patterns, and categorical self-perceived regulatory literacy. The second section comprised 11 objective multiple-choice questions mapped directly to primary statutory documents. These 11 items were structurally divided into two theory-driven competency domains:

1. **Declarative Regulatory Literacy (6 items)**: Assessing conceptual awareness, foundational prohibitions, and core professional boundaries.
2. **Procedural Regulatory Literacy (5 items)**: Assessing immediate administrative execution, statutory timelines, value limits, and operational liabilities.

Because the 11 objective items were designed to capture discrete, heterogeneous statutory regulations rather than a unified latent psychological trait, standard internal consistency metrics (e.g., Cronbach's alpha) were omitted as statistically inappropriate. Reliability was instead derived from direct statutory sourcing and expert consensus mapping. The study reporting adheres strictly to the Strengthening the Reporting of Observational Studies in Epidemiology guidelines (12).

### 2.5 Data collection and storage

Data collection was executed via a secure, encrypted survey platform. To protect participant privacy and minimize response bias regarding legal knowledge deficits, the protocol was designed to be completely anonymous. No personally identifiable information, institutional identification numbers, internet protocol addresses, or highly specific geographic identifiers were collected. Raw data matrices were transferred and stored exclusively on password-protected university servers, with access restricted solely to the primary research team. Data will be securely archived for five years post-publication in adherence to institutional research governance.

### 2.6 Ethical considerations

Ethical approval for this study was obtained from the regional research ethics committee, Qassim Providence (Approval No. 607-47-010651). Secure electronic informed consent was mandatory; the platform required participants to actively select “I Agree to Participate” on an introductory overview screen detailing the study's purpose, voluntary nature, and confidentiality metrics before unlocking the test items. No financial incentives or academic compensations were provided to eliminate coercion.

### 2.7 Statistical analysis

Data were analysed using Stata/SE version 16.1 software for Mac (StataCorp, College Station, TX, USA) (13). Socio-demographic characteristics, information channels, and educational training preferences were summarized using descriptive statistics (frequencies and percentages). To address the statistical dependence inherent in evaluating the same cohort of participants across two distinct competency domains, McNamar's test for paired nominal data was implemented to compare the proportion of correct vs. incorrect responses between the declarative and procedural domains. The binary competence passing benchmark was established *a priori* at ≥ 60%. This cut-off is anchored directly to standard criteria commonly enforced in health professions education and aligns with the national qualification baselines utilized for regional pharmacy licensure evaluations (14, 15). For participant-level analysis, a cumulative domain score of ≥ 60% was defined *a priori* as the minimum passing threshold. To determine the association between categorical self-perceived literacy (High vs. Low) and objective binary test performance (Pass vs. Fail), Fisher's exact test was utilized due to small, expected cell counts (<5) within the cross-tabulation matrix. All inferential assessments were two-tailed, with statistical significance defined at *p* < 0.05.

## 3 Results

### 3.1 Socio-demographic and practice characteristics

The final analytical sample consisted of 40 pharmacy interns, with a demographic distribution of 65.0% female (*n* = 26) and 35% male (*n* = 14). When identifying primary operational sources utilized to verify local pharmacy regulations, 75% (*n* = 30) cited the MOH, and 65% (*n* = 26) cited the SFDA. Crucially, 35% (*n* = 14) of emerging practitioners reported utilizing commercial generative artificial intelligence (AI) tools (e.g., ChatGPT) to resolve legal and regulatory questions, while 25% (*n* = 10) referenced internal pharmacy school documentation.

Before the objective evaluation, the majority of the cohort categorized their current knowledge of Saudi Arabian pharmacy regulations as “Fair” (52.5%, *n* = 21) or “Poor” (12.5%, *n* = 5), while 27.5% (*n* = 11) and 7.5% (*n* = 3) rated their baseline knowledge as “Good” or “Excellent,” respectively. Educational engagement interest was high, with 77.5% (*n* = 31) indicating a desire to attend dedicated regulatory training programs. The most preferred pedagogical delivery format was online courses (80.0%, *n* = 32), followed by in-person workshops (52.5%, (*n* = 21). The specific administrative areas highlighted by interns as most critical for future training included dispensing and controlled substances regulations (80.0%, *n* = 32), medication error reporting workflows (77.5%, *n* = 31), and institutional establishment and product registration requirements (75%, *n* = 30). Comprehensive socio-demographic indicators and behavioral characteristics are detailed in Table 1.

**Table 1:** Socio-Demographic and regulatory practice characteristics (*N* = 40).

Variable	*n* (%)
Gender Distribution	
Male	14 (35.0%)
Female	26 (65.0%)
Utilized Regulatory Information Channels *	
Ministry of Health (MOH) Portal	30 (75.0%)
Saudi Food and Drug Authority (SFDA) Portal	26 (65.0%)
Generative AI Tools (e.g., ChatGPT)	14 (35.0%)
Pharmacy School Internal Regulations	10 (25.0%)
Baseline Self-Perceived Regulatory Literacy	
Poor	5 (12.5%)
Fair	21 (52.5%)
Good	11 (27.5%)
Excellent	3 (7.5%)
Preferred Regulatory Training Topics^a^	
Dispensing and Controlled Substances Regulations	32 (80.0%)
Medication Error Reporting Systems	31 (77.5%)
Pharmaceutical Establishments & Product Registration	30 (75.0%)
Professional Licensing and Ethics	27 (67.5%)
Post-Market Surveillance and Compliance	27 (67.5%)
Primary Motivation for Training Attendance	
Career and Professional Advancement	19 (47.5%)
Personal Competency Development	10 (25.0%)
Maximizing Patient Safety	9 (22.5%)
Continuing Education Licensure Requirements	2 (5.0%)

a Multiple responses permitted.

### 3.2 Objective regulatory literacy assessment

Objective analysis revealed a profound bifurcation between declarative comprehension and procedural execution. Across the declarative sub-domain (6 items per participant; 240 pooled items), the cohort achieved an overall success rate of 80.0% (192/240 correct answers). Performance highlights included a 95% (*n* = 38) accuracy rate regarding statutory full-time Saudi pharmacy manager requirements, 87.5% (*n* = 35) for identifying the Saudi Commission for Health Specialties as the practitioner classification body, and 85% (*n* = 34) for both public media prescription advertising restrictions and controlled substance free-sample prohibitions. However, a significant institutional error occurred regarding local product registration: 50% (*n* = 20) incorrectly stated that all local products must hold United States Food and Drug Administration approval, whereas only 47.5% (*n* = 19) correctly identified the mandatory registration mandate with the sovereign SFDA.

In contrast, the procedural regulatory literacy sub-domain (5 items per participant; 200 pooled items) exposed severe systemic operational deficits, yielding an overall success rate of only 23.5% (47/200 correct answers). For fiscal administrative liability, only 10% (*n* = 4) correctly identified the pharmacy owner as legally responsible for product destruction costs of seized or counterfeit goods. For legal administrative timelines, only 45% (*n* = 18) identified the 5-year purchase invoice retention cycle, while identical minor proportions of 32.5% (*n* = 13) accurately identified the 30-day company response limit for practitioner information requests and the 5-year community pharmacy license renewal cycle. Remarkably, 0% (*n* = 0) of the interns were aware of the 100 Saudi Riyal maximum value limit for individual non-promotional materials given to healthcare professionals, with 57.5% (*n* = 23) operating under the false compliance assumption that no value cap exists if a company logo is displayed.

To model this gap without the statistical error of illegal item-pooling, a participant-level binary performance analysis (≥60%) vs. (Fail < 60%) was executed. At the participant level, the cohort demonstrated high proficiency within the declarative sub-domain, achieving an 80.0% overall success rate (95% CI: 64.4%–90.9%). Conversely, a significant operational performance gap emerged within the procedural sub-domain, where the overall success rate dropped to 22.5% (95% CI: 10.8%–38.5%; *χ*^2^ = 21.04, df = 1, *p* < 0.0001), confirming a severe knowledge-to-action deficit within the same cohort. Complete item statistics are categorized in Table 2.

**Table 2:** Item analysis of the two-domain regulatory literacy instrument (*N* = 40).

Declarative Sub-Domain (Basic Knowledge)			
Competency domain and statutory variable	Correct answer	Correct *n* (%)	Incorrect *n* (%)
Pharmacy Manager Status Mandate	Full-time Saudi^a^	38 (95.0%)	2 (5.0%)
Practitioner Licensing Body	Saudi Commission for Health Specialties^a^	35 (87.5%)	5 (12.5%)
Public Media Advertising Restriction	Advertising is restricted^a^	34 (85.0%)	6 (15.0%)
Free Sample Prohibitions	Controlled medications^a^	34 (85.0%)	6 (15.0%)
Prescription Only Medicines Sale	Requires a valid prescription^a^	32 (80.0%)	8 (20.0%)
Local Product Market Authorization Body	Registered with the SFDA^a^	19 (47.5%)	21 (52.5%)
**Procedural Sub-Domain (Operational Execution)**			
**Competency Domain and Statutory Variable**	**Correct Answer**	**Correct *n* (%)**	**Incorrect *n* (%)**
Invoice Retention Timelines	5 years after batch runs out^a^	18 (45.0%)	22 (55.0%)
Information Request Response Limits	30 days^a^	13 (32.5%)	27 (67.5%)
Facility License Renewal Cycles	Every five years^a^	12 (30.0%)	28 (70.0%)
Seized Product Destruction Liability	Pharmacy Owner^a^	4 (10.0%)	36 (90.0%)
Non-Promotional Material Value Cap	100 Saudi Riyals^a^	0 (0.0%)	40 (100.0%)

a Denotes correct statutory choice.

### 3.3 Predictive validity of self-perceived literacy

The secondary analysis evaluated the alignment between interns' self-perceived regulatory competency, High (Excellent/Good) vs. Low (Fair/Poor), and their objective overall binary performance across the entire 11-item tool. Cross-tabulation revealed a statistically significant predictive association evaluated via Fisher's exact test (*p* < 0.0001).

Among the interns who self-reported a High regulatory literacy level, 100% (*n* = 18/18) successfully achieved a passing score on the objective evaluation. Conversely, among those who self-reported a Low baseline literacy level, only 36.4% (*n* = 8/22) achieved a passing score, while 63.6% (*n* = 14/22) failed to reach the 60% objective benchmark. The complete paired dependency matrix is detailed in Supplementary Table 1 and Figure 2.

**Figure 2:**
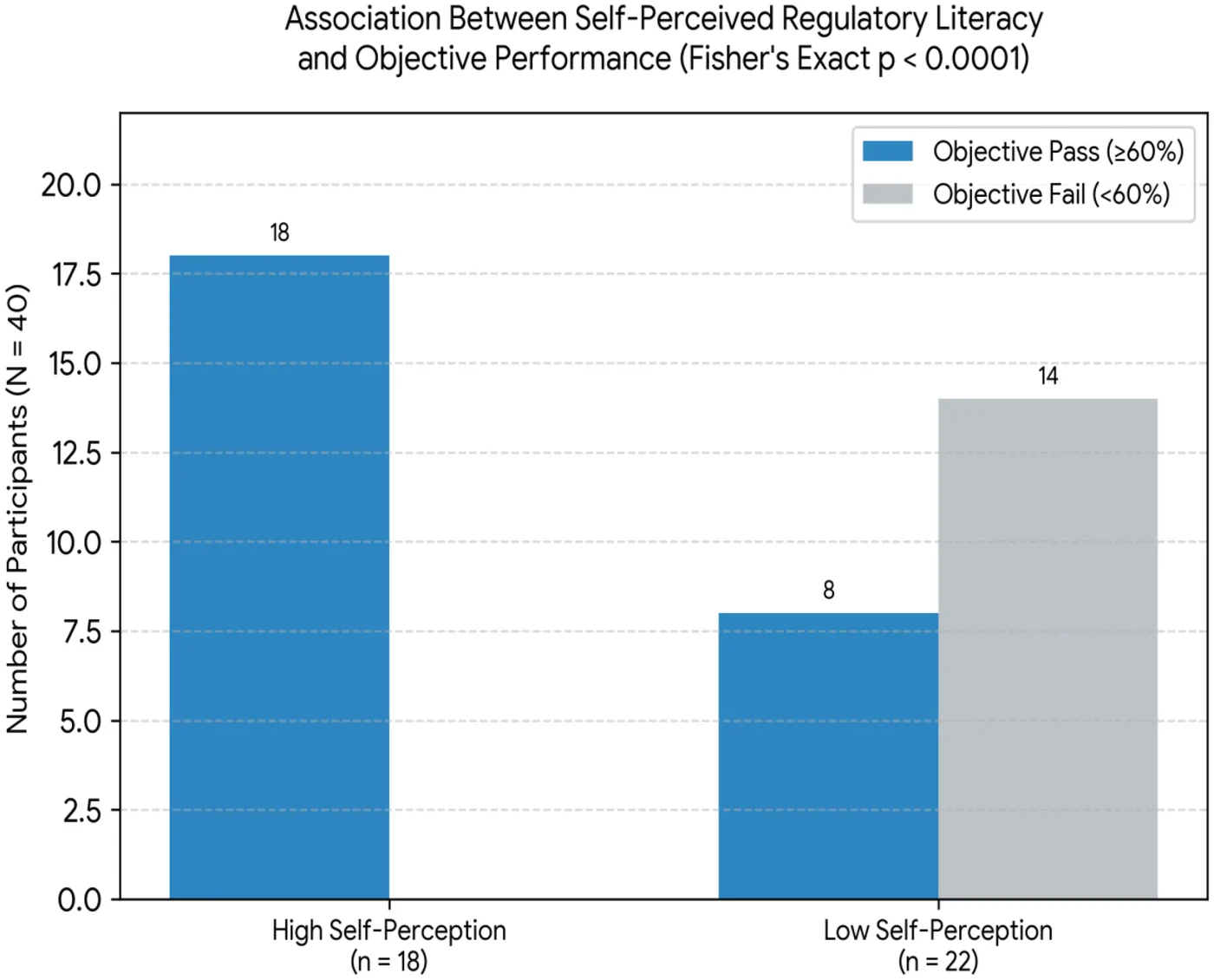
Association between baseline self-perceived regulatory literacy and overall objective performance outcomes (*N* = 40). Bars indicate the absolute number of pharmacy interns who achieved an objective passing score (≥60%) (blue) versus an objective failing score (< 60) (grey) when cross-tabulated against their initial self-reported literacy tiers (High/Confident vs. Low/Hesitant). While 100% (*n* = 18) of participants with high self-perception successfully passed the assessment, (63.6%) (*n* = 14) of those declaring low self-perception failed, exposing an acute awareness of under-preparedness. Statistical association and predictive calibration were evaluated using Fisher's exact test due to small cell frequencies (*n* < 5), yielding high statistical significance (two-tailed, *p* < 0.0001).

## 4 Discussion

This pilot study investigated how well pharmacy interns understand national basic and operational regulations before entering professional practice. The core finding reveals a clear gap between theoretical knowledge and practical execution. While interns demonstrate strong mastery of basic legal facts, their ability to execute operational administrative tasks drops significantly. To discuss these findings systematically, we first evaluate the cohort's baseline sociodemographic characteristics and information-seeking trends, before unpacking the statistical variations between their declarative understanding and practical performance gaps (Figure 3).

**Figure 3:**
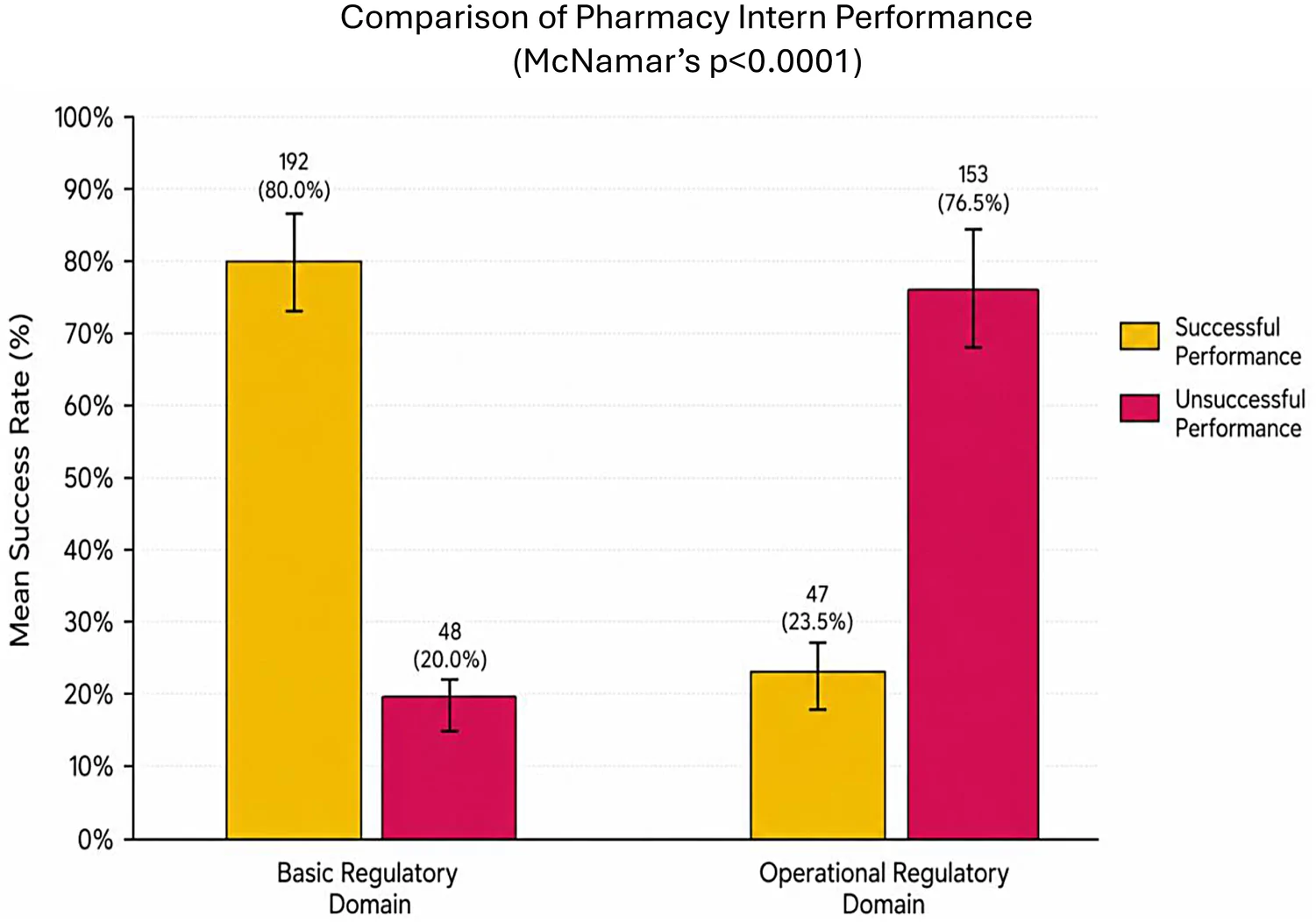
Comparison of pharmacy intern performance accuracy across declarative and procedural regulatory domains (*N* = 40). Bars represent the pooled percentage of successful (yellow) versus unsuccessful (pink) item responses across the Basic/Declarative Domain, 6 items per participant (*n* = 240) total pooled observations and the Operational/Procedural Domain, 5 items per participant (*n* = 200 total pooled observations, overall success rate of 23.5%). The profound performance inversion highlights a severe, high-risk knowledge-to-action (KTA) deficit. Statistical significance was evaluated at the participant binary outcome level (Pass ≥60%) vs. (Fail < 60%) utilizing McNamar's test to account for within-subject paired dependence (χ^2^ = 21.04, df = 1, *p* < 0.0001). Error bars denote 95% binomial confidence intervals. KTA, Knowledge-to-action; SFDA, Saudi Food and Drug Authority; MOH, Ministry of Health.

Rather than confirming ‘high metacognitive calibration' or advanced professional maturity, the strong alignment between low student self-perception and objective failure underscores an active state of under-preparedness. The most alarming empirical indicator of this educational gap is that 52.5% of final-year interns failed to identify their own regulatory authorization body (SFDA), with 50% explicitly misattributing local market authorization requirements to the United States Food and Drug Administration. This baseline vulnerability directly mirrors national trends documented by Alkayyal, Cheema, and Hadi (2017), who demonstrated that a striking 40.9% of senior Saudi pharmacy students could not correctly identify the SFDA as the primary national regulatory authority (8). While the internalization of international clinical standards ensures high-quality evidence-based practice, the complete omission of country-specific legal structures leaves emerging practitioners highly vulnerable when navigating sovereign market authorizations. In an aggressively nationalizing pharmacy sector under Saudi Vision 2030, confusing the foundational jurisdictional architecture of local marketing authorization introduces immediate compliance hazards and severe operational liabilities. This systemic blind spot is further corroborated by multi-center data from King Khalid University, showing that while declarative knowledge grows across academic levels, functional practice exposure regarding domestic regulatory workflows remains minimal (16).

Furthermore, the complete failure 0% of the cohort to identify statutory promotional value caps (100 Saudi Riyals), combined with the fact that 57.5% believed commercial logos exempt materials from regulatory oversight, reveals that entering practitioners are highly vulnerable to industry non-compliance. This procedural vacuum explains the high reliance (35%) on commercial generative AI tools (e.g., ChatGPT) to answer legal and regulatory queries. This finding directly aligns with recent data from a cross-sectional investigation among pharmacists in Riyadh, which revealed that 64.1% of practitioners actively maintain highly favourable attitudes toward integrating ChatGPT into their daily operations, despite minimal structured oversight. As highlighted in contemporary national administrative evaluations, this unverified reliance creates a dangerous compliance illusion (17, 18). Generative AI tools frequently synthesize conflicting international legal codes, lack hyper-localized statutory precision, and fluctuate in accuracy regarding SFDA updates. This creates compliance illusion, where an intern operates under the false assumption that an automated response is legally sound, when it violates Royal Decree No. M/108 and exposes the facility to severe penalties.

To bridge this theory-to-practice divide, theoretical jurisprudence must undergo an immediate paradigm shift within pharmacy education. Curricula must move beyond rote memorization of statutory text to integrate mandatory, simulation-based administrative modules. International validations of specialized, digital simulation networks have proven that virtual practice applications are essential to reinforce and recall complex pharmacy laws after traditional lectures conclude (19). This approach can directly fulfil the strategic mandate outlined by Saudi pharmacy educational boards, which explicitly calls for country-specific competency frameworks that abandon generalized curriculum standards in favour of hyper-localized, operational performance metrics (20). Interns should be required to navigate live SFDA electronic portals, execute mock facility license renewals, manage corporate liabilities for seized inventory, and audit pharmaceutical promotional materials against active national codes of conduct.

### 4.1 Methodological boundaries and study limitations

The insights derived from this investigation must be interpreted within several key parameters. First, the evaluation relies on self-reported operational practices, introducing vulnerability to recall and social desirability biases. Second, because the non-responding interns (*N* = 60) did not provide explicit consent, extracting their data for a comparative non-response bias analysis was ethically restricted under institutional protocols. Consequently, the localized nature of this single-center convenience sampling framework limits immediate widespread generalizability across alternative regional clusters. However, while the geographical scaling of this specific bilingual instrument beyond the immediate institutional network requires future verification, this pilot framework provides vital preliminary insights that serve as an empirical steppingstone for expansive, multi-center national evaluations to systematically map, generalize, and expand upon graduate regulatory literacy metrics across the Kingdom.

### 4.2 Operational recommendations

To translate these findings into structured educational strategies, a multi-tiered administrative paradigm shift is recommended:

- Doctor of Pharmacy curricula should transition from passive legal lectures to interactive simulations. Interns should be required to execute mock facility license renewals and navigate authentic SFDA electronic frameworks.
- Administrative coursework must explicitly differentiate international clinical guidelines from sovereign national workflows, emphasizing the distinct regulatory mandates of the SFDA and the MOH.
- Educational institutions should develop a centralized, SFDA-compliant student reference portal to counter the unverified and high-risk reliance on commercial AI systems.
- Future academic protocols should execute a multi-center national deployment of this content-validated pilot assessment framework to systematically identify and correct regional disparities in regulatory preparedness.

## 5 Conclusion

This pilot assessment study demonstrates that high declarative regulatory literacy among emerging pharmacists masks a notable deficit in procedural capability. To support graduates in becoming practice-ready and capable of upholding institutional compliance under Saudi Vision 2030, a structural paradigm shift within undergraduate pharmacy training is recommended. Rather than relying solely on theoretical jurisprudence coursework, pharmacy curricula could benefit from a transition toward experiential, simulation-based training frameworks. Based on these exploratory insights, we recommend that health professions educators consider integrating interactive, simulation-based operational modules into experiential training rotations to bridge the observed knowledge-to-action friction point.

## Data Availability

The original contributions presented in the study are included in the article/Supplementary Material, further inquiries can be directed to the corresponding author.
