# Supplementary material for: A two-domain regulatory literacy instrument among Saudi pharmacy interns: a single-center pilot assessment under vision 2030
Source: Front Med (Lausanne). 2026 Sep 23;13:1926745. doi: 10.3389/fmed.2026.1926745 (PMC13642119; doi:10.3389/fmed.2026.1926745)
Supplement: Supplementary file 1 [file Supplementaryfile1.pdf]

Appendix 1

The survey introduction

You are invited to participate in a research study to test a survey about pharmacy interns literacy of regulatory laws in the KSA. The goal is to ensure the survey is clear and functional before a national study. Participation is voluntary.

What Will I Do?

If you participate, you will complete an anonymous online questionnaire with demographic questions and 11 multiple-choice questions. It should take 10–15 minutes.

Confidentiality

Yes, your information is confidential and anonymous. We do not collect your name, ID, or IP address. Your answers cannot be traced back to you.

Right to Withdraw

Participation is voluntary. You can stop the survey at any point without penalty by closing your browser.

Questions

If you have questions, please contact the Principal Investigator at:

m.almutairi@qu.edu.sa

Consent

By clicking 'I Agree to Participate' below, you confirm you understand the study, agree to participate voluntarily, and agree to complete the questionnaire independently based purely on your own reflections.

- **I Agree to Participate] (Link to Survey)**
- **I Decline to Participate] (Exit Survey / Thank you screen)**

Section A – Demographics

Are you currently a final-year (graduate-year) pharmacy student or undertaking an internship at Qassim University?

☐ **Yes** (Continue to next questions)

☐ **No** (Thank the participant and end the questionnaire)

Gender: ☐ Male ☐ Female

What sources do you use to check pharmacy regulatory information (e.g., license renewal specifics, rules for free samples)? (Select all that apply)

☐ SFDA (Saudi Food and Drug Authority)

☐ MOH (Ministry of Health)

☐ My pharmacy school internal regulations

☐ AI (ChatGPT) for national regulations

☐ Others: ____________________

How would you rate your current knowledge regarding the pharmacy regulations in Saudi Arabia?

☐ Poor ☐ Fair ☐ Good ☐ Excellent

Are you interested in attending workshops or online training programs about pharmacy regulations in Saudi Arabia?

☐ Yes ☐ No

What is your preferred training format?

☐ In-person workshop ☐ Online course ☐ Short certification program ☐ Self-study

How frequently would you like to receive training on pharmacy regulations in Saudi Arabia?

☐ Once a year ☐ Twice a year ☐ Quarterly ☐ As needed

Which area would you like to be covered in training sessions? (Select all that apply)

☐ Professional Licensing and Ethics

☐ Pharmaceutical Establishments and Products Registration and Approval

☐ Post-Market Surveillance and Compliance

☐ Dispensing and Controlled Substances regulations

☐ Medication Error Reporting

☐ Other: ____________________

What motivates you the most to attend pharmacy regulations training programs in Saudi Arabia?

☐ Improving patient safety

☐ Personal development

☐ Career/Professional advancement

☐ Meeting continuing education requirements

☐ Other: ______________________

Section B: regulatory literacy assessment

Testing actual knowledge: This section assesses the pharmacist's intended *action* in real-world detection and management scenarios.

Please select the **best answer** for each question regarding the official regulations in Saudi Arabia.

**Regulation questions**	**Select your best answer**			
Who is responsible for the cost of destroying pharmaceutical products that are proven to be spoiled, counterfeit, expired, or health-damaging after being seized by the SFDA?	SFDA.	Ministry of Health.	Pharmacy owner.	The manufacturer.
How long must purchase invoices proving the source of products (including batch numbers) be kept by a pharmaceutical establishment?	Until the product is sold.	Permanently in digital format.	5 years after the batch runs out.	1 month after the batch runs out.
When a healthcare practitioner requests information about a pharmaceutical product from a company, what is the maximum time allowed for the company to provide this information?	Immediately.	30 days.	60 days.	7 days.
Is it permitted to advertise pharmaceutical products that require a medical prescription in public media (e.g., public newspapers, general websites)?	Yes, if approved by the MOH.	No, advertising is restricted.	Yes, if a physician provides a report.	Yes, if approved by the pharmacy manager.

What is the maximum value limit for a single "non-promotional promotional material" (e.g., a pen or notebook) that can be given to a healthcare practitioner?	50 Saudi Riyals.	500 Saudi Riyals.	No limit if it has a logo.	100 Saudi Riyals.
Which type of pharmaceutical product may *not* be provided as a free sample?	Herbal products.	OTC medicines.	New chemical entities.	Controlled medications.
What is the key regulatory distinction regarding the sale of "Prescription Only Medicines (POM)" in a community pharmacy?	Require separate, locked cabinet.	Require a valid prescription,	Require to be dispensed after 6 AM.	Require the pharmacist approval.
According to SFDA regulations, all pharmaceutical products sold must hold what specific status?	Pharmacy director approval.	Approved by the FDA.	Available in the market.	Registered with the SFDA.
What specific employment status and nationality are required for a pharmacy's technical manager?	Part-time KSA national.	Full-time non-KSA national.	Full-time KSA national.	Full-time expatriate.
Which official body is primarily responsible for classifying and licensing all practicing pharmacists in Saudi Arabia (before the MOH license is issued)?	Ministry of Health (MOH).	Saudi Food and Drug Authority (SFDA).	Saudi Pharmaceutical Society (SPS).	Saudi Commission for Health Specialties (SCFHS).
How often must a community pharmacy license be renewed in Saudi Arabia?	Annually.	Every two years.	Every five years.	Every 3 years.

Section C: Participant feedback

Thank you for completing the main sections of the questionnaire. As this is a regional study, your feedback on the survey design is essential for us to refine the tool for a future national study.

Please answer the following brief questions regarding your experience taking the survey:

1. **Approximately how many minutes did it take you to complete the entire questionnaire?**

…………………………………………………………………………………………… minutes

2. **Were the instructions provided at the beginning of each section clear and easy to follow?**

- ☐ Yes
- ☐ No

3. **Were any of the questions confusing, unclear, or difficult to understand?**

- ☐ Yes
- ☐ No

If yes, please specify which question(s) and briefly explain why they were unclear:

Question number ……………………………………………………………………………………..

4. **Do you feel the topics covered in the knowledge aassessment are relevant to your future practice as a licensed pharmacist in Saudi Arabia?**

- ☐ Highly Relevant
- ☐ Relevant
- ☐ Neutral
- ☐ Not Relevant
- ☐ Highly Irrelevant

5. **Do you have any other general comments or suggestions for improving the clarity, length, or content of this questionnaire?**

……………………………………………………………………………………………………….

Thank you for your valuable input and participation!

مقدمة

أنت مدعو للمشاركة في دراسة بحثية تجريبية لاختبار استبانة حول معرفة طلاب السنة النهائية في الصيدلة باللوائح والأنظمة في المملكة العربية السعودية. الهدف هو التأكد من وضوح الاستبانة وفعاليته قبل إطلاقه على المستوى الوطني.

المشاركة طوعية بالكامل.

ماذا سأفعل؟

إذا وافقت على المشاركة، ستكمل استبياناً مجهول الهوية عبر الإنترنت يحتوي على أسئلة ديموغرافية و 11 سؤال معرفي متعدد الخيارات. ينبغي أن يستغرق ذلك **10إلى 15 دقيقة** من وقتك.

السرية التامة للمعلومات

نعم، معلوماتك سرية ومجهولة تماماً. نحن لا نجمع اسمك، أو رقمك الجامعي، أو عنوان IP الخاص بك. لا يمكن ربط إجاباتك بهويتك.

الحق في الانسحاب

المشاركة طوعية. يمكنك التوقف عن إكمال الاستبانة في أي لحظة دون عقوبة بمجرد إغلاق نافذة المتصفح.

للأسئلة والاستفسارات

إذا كانت لديك أسئلة، يرجى التواصل مع الباحث الرئيسي على:

m.almutairi@qu.edu.sa

الموافقة

"بالنقر على زر "أوافق على المشاركة "أدناه، فإنك تؤكد أنك تفهم الدراسة، وتوافق على المشاركة طواعية، وتوافق على إكمال الاستبيان بشكل مستقل بناءً على آرائك الشخصية وحدها

- **أوافق على المشاركة** (رابط يؤدي إلى الاستبانة)
- **لا أوافق على المشاركة** (خروج من الاستبانة)

القسم ١ – البيانات الديموغرافية

هل أنت حالياً طالب صيدلة في السنة النهائية (سنة التخرج) أو تقوم بفترة تدريب (امتياز) في جامعة القصيم؟

- ☐نعم (يرجى المتابعة إلى الأسئلة التالية)
- ☐لا (شكراً لمشاركتك، ينتهي الاستبيان هنا)

الجنس:

- ☐ذكر
- ☐أنثى

ما هي المصادر التي تستخدمها للتحقق من المعلومات التنظيمية الصيدلانية (مثل: تفاصيل تجديد الترخيص، القواعد الخاصة بالعينات المجانية؟) (يرجى اختيار كل ما ينطبق)

- ☐هيئة الغذاء والدواء السعودية(SFDA)
- ☐وزارة الصحة(MOH)
- ☐اللوائح الداخلية لكلية الصيدلة الخاصة بي
- ☐الذكاء الاصطناعي (ChatGPT) للوائح الوطنية
- ☐مصادر أخرى ______________________ :

كيف تُقيّم معرفتك الحالية باللوائح والأنظمة الصيدلانية في المملكة العربية السعودية؟

- ☐ضعيفة
- ☐مقبولة (متوسطة)
- ☐جيدة
- ☐ممتازة

هل أنت مهتم بحضور ورش عمل أو برامج تدريبية عبر الإنترنت حول اللوائح الصيدلانية في المملكة العربية السعودية؟

- ☐نعم
- ☐لا

ما هو تنسيق التدريب المفضل لديك؟

- ☐ورشة عمل حضورية
- ☐دورة عبر الإنترنت
- ☐برنامج شهادة قصير
- ☐دراسة ذاتية

كم مرة تفضل الحصول على تدريب حول اللوائح الصيدلانية في المملكة العربية السعودية؟

- ☐مرة واحدة في السنة
- ☐مرتين في السنة
- ☐كل ثلاثة أشهر (ربع سنوية)
- ☐عند الحاجة

أي مجال (مجالات) تفضل تغطيته في الدورات التدريبية؟)يرجى اختيار كل ما ينطبق(

- ☐التراخيص المهنية وآداب المهنة
- ☐تسجيل واعتماد المؤسسات والمنتجات الصيدلانية
- ☐مراقبة ما بعد التسويق والامتثال للوائح
- ☐لوائح صرف الأدوية والمواد الخاضعة للرقابة
- ☐الإبلاغ عن الأخطاء الدوائية
- ☐مجال آخر________________________ :

ما الذي يحفزك أكثر لحضور برامج تدريبية حول اللوائح الصيدلانية في المملكة العربية السعودية؟

- ☐تحسين سلامة المرضى
- ☐التطوير الشخصي
- ☐التقدم الوظيفي/المهني
- ☐تلبية متطلبات التعليم المستمر
- ☐دوافع أخرى________________________ :

القسم٢: تقييم المعرفة

**الاسالة النظامية**	**اختر الإجابة الصحيحة**			
**من يتحمل تكلفة إتلاف المنتجات الصيدلانية التي يثبت أنها فاسدة، أو مغشوشة، أو منتهية الصلاحية أو ضارة بالصحة بعد مصادرتها؟**	هيئة الغذاء والدواء السعودية (SFDA)	وزارة الصحة	مالك الصيدلية	الشركة المُصنِّعة
**كم مدة الاحتفاظ بفواتير الشراء التي تثبت مصدر المنتجات (بما في ذلك أرقام الشغيلات/الدفعات) في المنشأة الصيدلانية؟**	شهر واحد	5 سنوات.	بشكل دائم بصيغة رقمية.	حتى يتم بيع المنتج
**عندما يطلب ممارس الرعاية الصحية معلومات حول منتج صيدلاني من شركة ما، ما هو الحد الأقصى للوقت المسموح به للشركة لتقديم هذه المعلومات؟**	7 أيام	60 يوماً	30 يوماً	فوراً
**هل يُسمح بالإعلان عن المنتجات الصيدلانية التي تتطلب وصفة طبية في وسائل الإعلام العامة (مثل الصحف العامة والمواقع الإلكترونية العامة)؟**	نعم، إذا وافق مدير الصيدلية.	نعم، إذا قدم الطبيب تقريراً.	لا، الإعلان مقيّد.	نعم، إذا وافقت وزارة الصحة.
**ما هو الحد الأقصى للقيمة المسموح بها لمادة ترويجية واحدة "غير ترويجية" (مثل قلم أو دفتر ملاحظات) يمكن تقديمها لممارس رعاية صحية؟**	100 ريال سعودي	لا يوجد حد إذا كان يحمل شعاراً.	500 ريال سعودي	50 ريال سعودي
**أي نوع من المنتجات الصيدلانية لا يجوز تقديمه كعينة مجانية؟**	أدوية خاضعة للرقابة	كيانات كيميائية جديدة	الأدوية اللاوصفية	منتجات عشبية
**ما هو التمييز التنظيمي الرئيسي المتعلق ببيع "الأدوية التي تصرف بوصفة طبية فقط (POM)" في صيدلية؟**	تتطلب موافقة الصيدلي.	تتطلب صرفها بعد الساعة 6 صباحاً.	تتطلب وصفة صالحة من ممارس صحي مرخص.	تتطلب خزانة منفصلة ومغلقة.
**وفقًا لأنظمة هيئة الغذاء والدواء السعودية، يجب أن تتمتع جميع المنتجات الصيدلانية المباعة بأي حالة محددة؟**	موافقة مدير الصيدلية	معتمدة من هيئة الغذاء والدواء	متوفرة في السوق	مسجلة لدى هيئة الغذاء والدواء السعودية

**ما حالة التوظيف والجنسية المطلوبة لمدير الشؤون الفنية في الصيدلية؟**	دوام جزئي – سعودي	دوام كامل – غير سعودي	دوام كامل – سعودي	دوام كامل – وافد
**ما الجهة الرسمية المسؤولة بشكل أساسي عن تصنيف وترخيص جميع الصيادلة الممارسين في المملكة العربية السعودية (قبل إصدار ترخيص وزارة الصحة)؟**	وزارة الصحة (MOH	هيئة الغذاء والدواء السعودية (SFDA)	الجمعية الصيدلانية السعوديةSPS)	الهيئة السعودية للتخصصات الصحية (SCFHS)
**كم مرة يجب تجديد ترخيص صيدلية المجتمع في المملكة العربية السعودية؟**	سنويًا	كل سنتين	كل خمس سنوات	كل ثلاث سنوات

القسم الثالث: الملاحظات حول الدراسة التجريبية (الاستطلاعية)

نشكرك على إكمال الأقسام الرئيسية من الاستبانة. نظراً لأن هذه دراسة تجريبية (استطلاعية)، فإن ملاحظاتك حول تصميم الاستبانة ضرورية بالنسبة لنا لتحسين الأداة قبل استخدامها في دراسة وطنية مستقبلية.

يرجى الإجابة على الأسئلة الموجزة التالية بخصوص تجربتك في الإجابة على الاستبانة:

كم دقيقة استغرقت تقريباً لإكمال الاستبانة بأكمله (القسمين الأول والثاني)؟

....دقيقة

هل كانت التعليمات المقدمة في بداية كل قسم واضحة وسهلة المتابعة؟

☐نعم

☐لا

هل كانت أي من الأسئلة في قسم "تقييم المعرفة غير واضحة، أو صعبة الفهم؟

☐نعم

☐لا

إذا كانت الإجابة نعم، يرجى تحديد رقم السؤال (الأسئلة) وشرح موجز لسبب عدم وضوحها:

رقم السؤال.. ....

هل تشعر أن المواضيع التي تمت تغطيتها في "تقييم المعرفة" ذات صلة بممارستك المستقبلية كصيدلي مرخص في المملكة العربية السعودية؟

☐وثيقة الصلة جداً

☐ذات صلة

☐محايد

☐غير ذات صلة

☐غير ذات صلة على الإطلاق

هل لديك أي تعليقات عامة أخرى أو اقتراحات لتحسين وضوح هذا الاستبانة، أو طوله، أو محتواه؟

تعليقات.. .... :

شكراً جزيلاً على مساهمتك القيمة ومشاركتك!
