# Supplementary material for: A two-domain regulatory literacy instrument among Saudi pharmacy interns: a single-center pilot assessment under vision 2030
Source: Front Med (Lausanne). 2026 Sep 23;13:1926745. doi: 10.3389/fmed.2026.1926745 (PMC13642119; doi:10.3389/fmed.2026.1926745)
Supplement: Supplementary file 2 [file Table1.docx]

| **Supplementary Table 1.** Contingency Analysis of Self-Perceived vs. Objective Regulatory Performance | | | | |
| --- | --- | --- | --- | --- |
| **Baseline Self-Perception** | **Objective Pass**  ($\geq$60%) | **Objective Fail**  (< 60%) | **Total Participants**  n | **Fisher's Exact Test**  p-value |
| **High** *(Excellent/Good)* | 18 (45%) | 0 (0%) | 18 | p < 0.0001 |
| **Low** *(Fair/Poor)* | 8 (20%) | 14 (35%) | 22 |  |
| **Total Cohort** | 26 (65%) | 14 (35%) | 40 |  |
